# Peripheral perfusion index as a predictor of reintubation in critically ill surgical patients

**DOI:** 10.1186/s12871-024-02608-4

**Published:** 2024-07-09

**Authors:** Ayman Abougabal, Ahmed Hasanin, Marwa Abdel-Fatah, Maha Mostafa, Ahmed A. Ismail, Sara Habib

**Affiliations:** Department of Anesthesia and Critical Care Medicine, Cairo University, Cairo, Egypt

**Keywords:** Mechanical ventilation, Weaning, Extubation, Reintubation, Peripheral perfusion index, Spontaneous breathing trial

## Abstract

**Purpose:**

We aimed to evaluate the ability of the peripheral perfusion index (PPI) to predict reintubation of critically ill surgical patients.

**Methods:**

This prospective observational study included mechanically ventilated adults who were extubated after a successful spontaneous breathing trial (SBT). The patients were followed up for the next 48 h for the need for reintubation. The heart rate, systolic blood pressure, respiratory rate, peripheral arterial oxygen saturation (SpO_2_), and PPI were measured before-, at the end of SBT, 1 and 2 h postextubation. The primary outcome was the ability of PPI 1 h postextubation to predict reintubation using area under the receiver operating characteristic curve (AUC) analysis. Univariate and multivariate analyses were performed to identify predictors for reintubation.

**Results:**

Data from 62 patients were analysed. Reintubation occurred in 12/62 (19%) of the patients. Reintubated patients had higher heart rate and respiratory rate; and lower SpO_2_ and PPI than successfully weaned patients. The AUC (95%confidence interval) for the ability of PPI at 1 h postextubation to predict reintubation was 0.82 (0.71–0.91) with a negative predictive value of 97%, at a cutoff value of ≤ 2.5. Low PPI and high respiratory rate were the independent predictors for reintubation.

**Conclusion:**

PPI early after extubation is a useful tool for prediction of reintubation. Low PPI is an independent risk factor for reintubation. A PPI > 2.5, one hour after extubation can confirm successful extubation.

## Introduction

Despite the continuous improvement of its protocols, discontinuation of mechanical ventilation remains a challenging decision for intensivists. Clear guidelines are present for evaluation of patients before extubation [1, 2]; nevertheless, there is still a non-negligible number of patients who fail after extubation [3]. The need to reinstitute ventilatory support in patients who were recently extubated is associated with a considerable risk of mortality [3, 4]. This increased mortality has many causes, one of them is the clinical deterioration between extubation and re-institution of mechanical ventilation [3, 4]. Late initiation of non-invasive mechanical ventilation after confirmed failed extubation is not encouraged, and is associated with poor outcomes [5]. Therefore; early detection of weaning failure after extubation would allow adequate assessment of the patient, better diagnosis of the cause of failure, and timely initiation of the supportive respiratory measures [6].

Switching the patient from positive pressure ventilation to spontaneous breathing is usually associated with increased oxygen demands which sometimes exceed oxygen delivery [7]. The increase in work of breathing during spontaneous breathing diverts blood flow from peripheral tissue towards the diaphragm and other respiratory muscles and reduces oxygen delivery to the peripheral tissues [8, 9]. Therefore, reduced peripheral tissue perfusion was reported in patients with failed weaning from mechanical ventilation [10, 11]. Plethysmography-derived peripheral perfusion index (PPI) is measured non-invasively and reflects the ratio between pulsatile and non-pulsatile components of peripheral circulation. PPI is influenced by two factors: the cardiac output and the balance between the sympathetic and parasympathetic tone. Therefore, PPI is used as a surrogate index for the sympathetic tone as it usually decreases in cases of stress, pain, and sympathetic stimulation [12]. PPI before extubation had been previously found predictive of failed weaning [13]; however, there is lack of data regarding its ability to predict reintubation after successful spontaneous breathing trial (SBT) and extubation. We hypothesize that impairment of PPI after extubation might be predictive of reintubation.

## Patients and methods

This prospective observational study was conducted in Cairo University Hospital’s Surgical Intensive Care Unit (SICU) between October 2022 to April 2023 after institutional Ethics Committee approval (MS-398-2022). Written informed consent was obtained from patient’s guardian before enrolment.

We consecutively included adult (> 18 years) mechanically ventilated patients for > 48 h who were extubated following a successful SBT. Patients with known history of peripheral vascular disease, hand injuries or infection that preclude the application of the device probe were excluded.

Readiness of weaning from mechanical ventilation was judged by the attending intensivist in accordance with the local protocol which included: resolution of the original cause of mechanical ventilation, adequate cough (defined as the presence of adequate cough upon temporary discontinuing of mechanical ventilation and asking the patient to cough as strong as possible), absence of excessive tracheobronchial secretions (the need for frequent endotracheal suctioning), adequate oxygenation (PaO_2_ > 60 mmHg at positive end-expiratory pressure of ≤ 8 cmH_2_O, and FIO_2_ ≤ 0.5), *p*H and PaCO_2_ appropriate for patients’ baseline respiratory status, and stable cardiovascular status [1, 2]. The cuff leak test was performed in high-risk patients for upper airway edema as judged by the attending intensivist.

The SBT was performed via the pressure support mode (pressure support of 5 cmH_2_O and positive end expiratory pressure of 5 cmH_2_O). After 30 min, the weaning parameters were re-evaluated. The attending intensivist was responsible for the decision of extubation and was blinded to the PPI measurements.

The SBT was considered a failure when one or more of the following criteria were present: altered consciousness, respiratory rate > 35 breath/minute, increased work of breathing (use of accessory respiratory muscle, asynchronous chest-abdominal respiratory movement, intercostal retraction), SpO_2_ < 90% or PaO_2_ < 60 mmHg on FiO_2_ of 0.4, hemodynamic instability (heart rate > 140 bpm, systolic blood pressure > 180 mmHg or < 90 mmHg, or > 20% change from baseline reading in both) [1, 2]. Patients with failed SBT were excluded from the study. Patients with successful SBT were extubated and followed up for the next 48 h for the need for reintubation. Simple oxygen mask was provided to maintain SpO_2_ > 92%.

The PPI was measured using Radical-7 device pulse co-oximeter (Masimo Corporation, Irvine, CA). The probe was covered with an opaque shield to avoid the external light effect. The PPI readings were recorded before and at the end of the SBT, then at 1 and 2 h postextubation. We recorded the average of the highest and lowest PPI during a 30 s period.

The systolic blood pressure, heart rate, respiratory rate, and SpO_2_ were measured at the same intervals as the PPI readings.

The primary outcome was the ability of the PPI at 1 h postextubation to predict reintubation within 48 h from extubation.

The Secondary outcomes included the ability of the PPI before the SBT, at the end of the SBT and at 2 h postextubation, to predict reintubation within 48 h from extubation.

Other outcomes included: PPI, systolic blood pressure, heart rate, respiratory rate, and SpO2 at the prespecified intervals, arterial blood gases before onset of the SBT, rapid shallow breathing index (respiratory rate/tidal volume) at the end of the SBT. Demographic data, comorbidity, the cause of mechanical ventilation, Acute Physiology and Chronic Health Evaluation (APACHE) II (data for the APACHE II score were collected at the time of ICU admission), days of mechanical ventilation, and time to reintubation were also recorded.

### Sample size

The sample size was calculated using MedCalc Software version 14 (MedCalc Software bvba, Ostend, Belgium) to detect an area under receiver operating characteristic curve (AUC) of 0.8 and the null hypothesis was set at 0.5. Assuming that the incidence of reintubation is 20% [2], a minimum number of 55 patients of (with at least 11 patients needing reintubation) was required to achieve a study power of 90% and an alpha error of 0.05.

### Statistical analysis

Analysis of the data was performed using Statistical package for social science (SPSS) software, version 26 for Microsoft Windows (IBM Corp. NY, USA). The patients were divided according to the need for reintubation within 48 h from extubation into successful weaning group and reintubation group. Categorical data are reported as frequency (percentages) and were analysed using the Chi-squared or Fisher’s exact as fitting. Continuous data were assessed for normality using the Kolmogorov-Smirnov test. Normally distributed data are reported as means ± standard deviations and were analysed using the student t-test. Skewed data are reported as medians (quartiles) and were analysed using the Mann-Whitney U test. Repeated measured data were analysed using the repeated measure analysis of variance. The Bonferroni test was used for adjustment for multiple testing. The AUC analysis was performed to evaluate the ability of PPI to predict reintubation within 48 h postextubation. The best cutoff value was identified using the Youden index, and its sensitivity, specificity, positive and negative predictive values are reported. The respiratory and hemodynamic parameters as well as the PPI at 1 h postextubation were included into a multivariate analysis model with forward stepwise selection method to determine the independent predictors for reintubation. A *P*-value < 0.05 was considered significant.

## Results

**Fig. 1:**
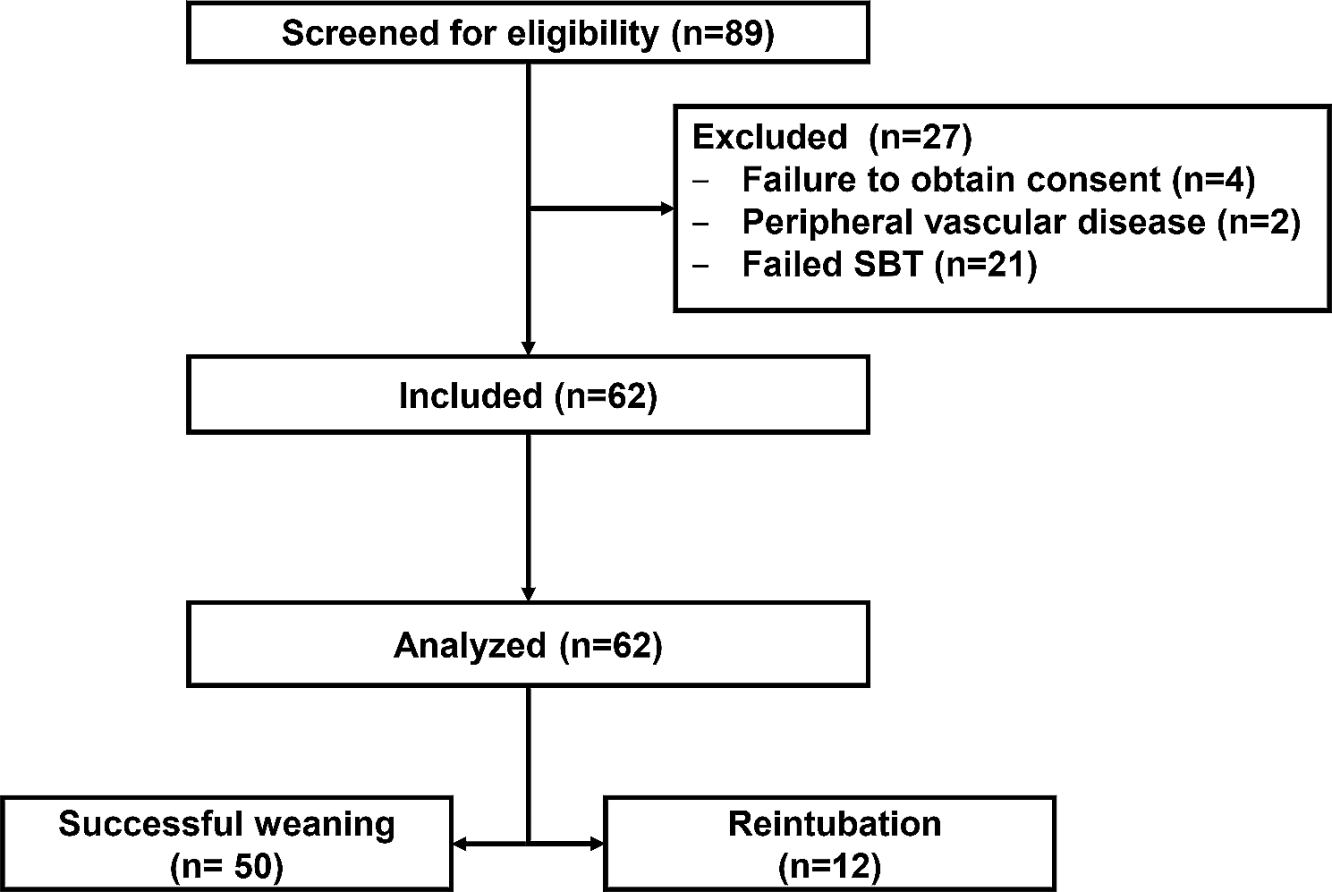
Patients’ enrolment. SBT: spontaneous breathing trial

Eighty-nine patients were screened for eligibility, six patients were excluded for not fulfilling of the inclusion criteria, 21 patients failed during the SBT, and 62 patients were extubated. The number of patients needing reintubation was 12/62 (19%) with a median (quartiles) time to reintubation of 5 (4, 24) h from the extubation (Fig. 1). The causes of reintubation included respiratory failure (10/12 [83%]), hemodynamic instability (1/12 [8%]), and disturbed conscious level (1/12 [8%])

Demographic, clinical, and laboratory data in both successful weaning- and reintubation groups are presented in Table 1.

**Table 1:** Demographic and respiratory data. Data are presented as mean ± standard deviation, median (quartiles), and frequency (%)

	Successful weaning (*n* = 50)	Reintubation (*n* = 12)	*P*-value
Age (years)	35 (29, 54)	28 (23, 42)	0.112
Male sex	16 (32%)	2 (17%)	0.293
Body mass index (kg/m^2^)	29 (27, 33)	27 (25, 33)	0.150
Comorbidity			
Hypertension	12 (24%)	4 (33%)	0.507
Ischemic heart disease	2 (4%)	0 (0%)	1.000
Atrial fibrillation	0 (0%)	1 (8%)	0.194
COPD	2 (4%)	0 (0%)	1.000
Bronchial asthma	1 (2%)	0 (0%)	1.000
Diabetes mellitus	7 (14%)	1 (8%)	0.599
APACHE II score	9 ± 6	11 ± 7	0.226
Norepinephrine infusion rate (mcg/kg/min)	0.08 (0.06, 0.13)	0.09 (0.02, 0.19)	0.668
PaO_2_/FiO_2_ ratio	331 (261, 377)	255 (234, 325)	0.012
PaCO_2_ (mmHg)	36 ± 6	36 ± 4	0.928
pH	7.42 ± 0.07	7.39 ± 0.06	0.177
HCO_3_ (mmol/L)	24 ± 4	22 ± 4	0.092
RSBI at the end of SBT	74 ± 20	82 ± 30	0.213
Lactate (mmol/L)	1.6 (1.3, 2.2)	2.2 (1.5, 2.9)	0.141
Cause of ICU admission Peripartum haemorrhage Severe preeclampsia/eclampsia Complicated abdominal surgery Soft tissue sepsis Post-abdominal exploration Surgery for major trauma Major head and neck surgery Neurosurgery Chest infection	11 (22%) 9 (18%) 8 (16%) 7 (14%) 7 (12%) 3 (6%) 2 (4%) 2 (4%) 1 (2%)	2 (17%) 5 (42%) 2 (17%) 0 (0%) 0 (0%) 1 (8%) 0 (0%) 1 (8%) 1 (8%)	0.531
Cause of mechanical ventilation Hemodynamic instability Respiratory failure Laryngeal oedema	27 (54%) 22 (44%) 1 (2%)	5 (42%) 7 (58%) 0 (0%)	0.701
Days of mechanical ventilation	3.0 (2.0, 3.3)	3.0 (2.3, 4.0)	0.123

APACHE: Acute Physiology and Chronic Health Evaluation, COPD: chronic obstructive pulmonary disease, RSBI: shallow rapid breathing index, SBT: spontaneous breathing trial

**Fig. 2:**
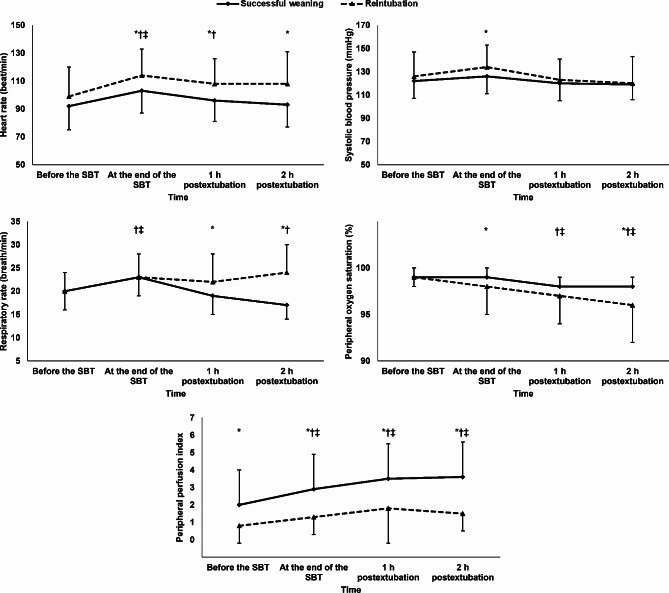
Repeated measures for hemodynamic and respiratory parameters. Markers are means and error bars are standard deviations. *denotes significance between the groups, †denotes significance in relation to pre-SBT reading within the successful weaning group, ‡ denotes significance in relation to the pre-SBT reading within the reintubation group. SBT: spontaneous breathing trial

Patients in the reintubation group had higher heart rate and respiratory rate, and lower SpO_2_, and PPI than those in the successful weaning group after extubation (Fig. 2). The multivariate analysis (including hemodynamic, respiratory data and PPI at 1 h postextubation) showed that low PPI and high respiratory rate were the independent predictors for reintubation (Table 2).

**Table 2:** Univariate and multivariate analysis for hemodynamic and respiratory parameters 1 h postextubation for prediction of reintubation

Univariate analysis		
	Odds ratio (95% CI)	P value
Heart rate	1.05 (1.00-1.20)	0.036
Systolic blood pressure	1.01 (0.97–1.04)	0.692
SpO_2_	0.80 (0.61–1.03)	0.084
Respiratory rate	1.19 (1.03–1.39)	0.022
PPI	0.46 (0.25–0.84)	0.013
Multivariate analysis		
	Odd ratio (95% CI)	P value
Respiratory rate	1.25 (1.03–1.52)	0.024
PPI	0.47 (0.25–0.87)	0.016

CI: confidence interval, PPI: peripheral perfusion index, SpO_2_: peripheral oxygen saturation

**Fig. 3:**
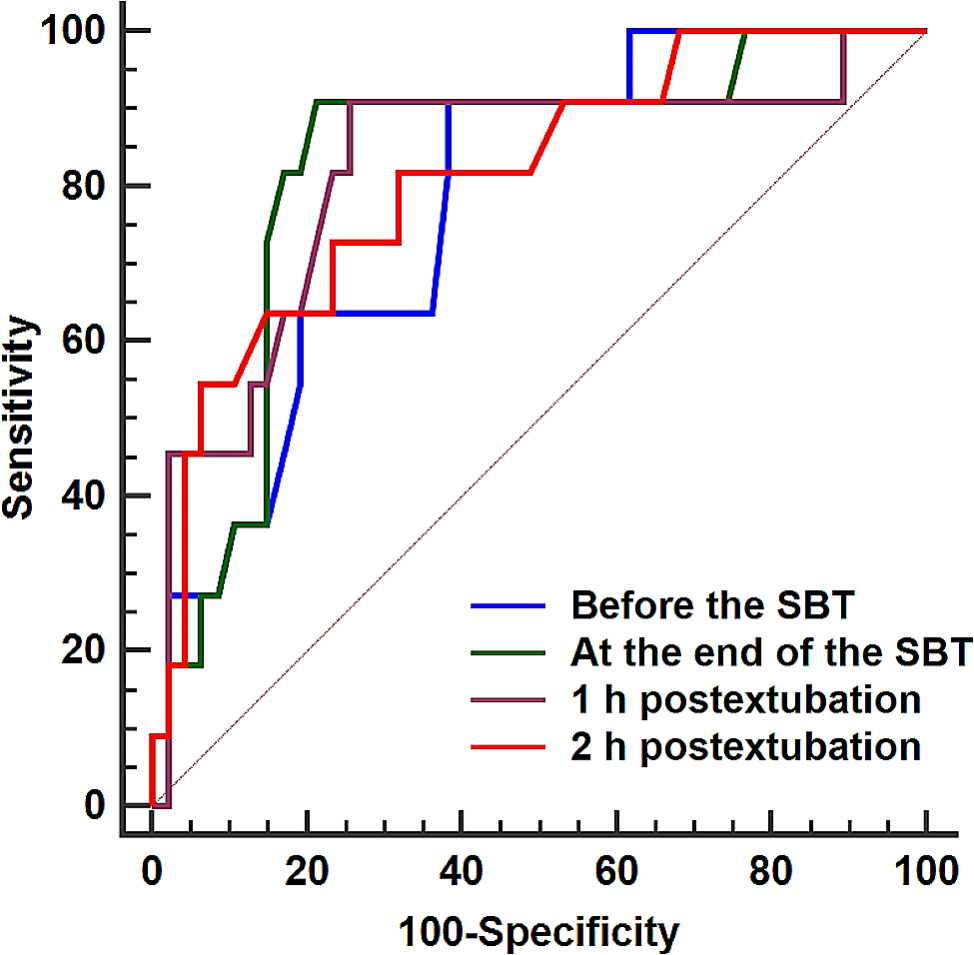
Receiver operating characteristic curves for the ability of the peripheral perfusion index to predict reintubation at different timepoints. SBT: spontaneous breathing trial

At 1 h postextubation, the AUC (95% confidence interval) for PPI was 0.82 (0.71–0.91); and a PPI value of ≤ 2.5 had a negative predictive value of 97% (Table 3). The AUCs for the PPI at the four timepoints were comparable. (Table 3) (Fig. 3).

**Table 3:** The AUC analysis for the ability of PPI to predict the reintubation

	AUC (95% CI)	Sensitivity % (95% CI)	Specificity% (95% CI)	PPV% (95% CI)	NPV% (95% CI)	Cut-off value
Before the SBT						
PPI	0.78 (0.65–0.89)	82 (48–98)	62 (46–76)	33 (17–54)	93 (79–99)	≤ 1.3
RR	0.53 (0.39–0.65)	83 (52–98)	28 (16–43)	22 (11–36)	88 (62–98)	≤ 22 breath/min
At the end of SBT						
PPI	0.83 (0.71–0.91)	92 (62–100)	76 (62–87)	48 (27–69)	97 (87–100)	≤ 1.9
RR	0.52 (0.39–0.65)	75 (43–95)	40 (26–55)	23 (11–39)	87(66–97)	≤ 24 breath/min
After 1 h of extubation						
PPI	0.82 (0.71–0.91)	92 (62–100)	74 (60–84)	46 (26–67)	97 (86–100)	≤ 2.5
RR	0.68 (0.55–0.80)	100(73–100)	36 (23–51)	27 (15–43)	100(82–100)	> 16 breath/min
After 2 h of extubation						
PPI	0.80 (0.68–0.89)	83 (52–98)	64 (49–77)	36 (19–56)	94 (80–99)	≤ 2.8
RR	0.82 (0.71–0.91)	83 (52–98)	78 (64–89)	48 (26–70)	95(84–99)	> 19 breath/min

*The P-value of the comparison between the PPI’s and RR’s AUC at each time point was: 0.052, 0.006, 0.165, 0.821, respectively. AUC: area under receiver operating characteristic curve, CI: confidence interval, NPV: negative predictive value, PPV: positive predictive value, PPI: peripheral perfusion index, RR: respiratory rate

The AUC for repiratory rate was statistically significant for prediction of reintubation only at 1 and 2 h after extubation and it was comparable to the that of the PPI at the same timepoints (Table 3). However, the PPI had higher specificity and positive predictive value than the respiratory rate (Table 3).

## Discussion

We report that PPI, obtained during and after disconnection from the mechanical ventilation has excellent ability to predict reintubation within 48 h. Furthermore, post-extubation PPI and respiratory rate were the two independent predictors for reintubation. The PPI is a measure of the ratio between the pulsatile and non-pulsatile components of the peripheral circulation and is influenced by two factors: alteration of the balance between the sympathetic and parasympathetic tone; and cardiac output [14, 15]. Failed extubation is associated with stress response and sympathetic stimulation which alters the values of the PPI [10, 11] and this explains the relation between low PPI and reintubation in the current study.

Switching patients from positive to negative pressure ventilation is associated with increased venous return and a corresponding increase in cardiac output [16–18]. Thus, it is expected to have increased PPI values during and after the SBT since the PPI is influenced by the change in cardiac output.

A previous study had evaluated the change in PPI during the weaning process of 44 surgical critically ill patients. Lotfy et al. reported that the PPI increases during the SBT, and that failure of the PPI to increase during the SBT is associated with an increased probability of failed weaning [13]. The current study supports the previous report, as we found that PPI increases during the SBT and further increases after extubation. We also found an association between low PPI values and reintubation. However, major differences are present between the current study and the previous study in terms of the type of patients and primary outcome. The current study included patients after extubation to predict reintubation, while Lotfy et al. included patients during the SBT, and defined failed weaning as reintubation or failure by the end of the SBT. Therefore, 40% of failed patients in the previous study had failed the SBT and were not extubated. Hence, our study adds more insights to previous literature as it focused on reintubation which is associated with several hemodynamic and airway hazards.

Reintubation is a serious situation in critical care practice and is an independent risk factor for poor patient outcomes [3, 4]. Early prediction of such condition could alarm the intensivist to initiate measures to detect and correct the causes of failure. Furthermore, early prediction of reintubation could enhance early initiation of non-invasive respiratory support. There is a growing interest for the use of early respiratory support after extubation, through either non-invasive ventilation [19] high-flow nasal cannula [20], or both [21], to decrease the incidence of reintubation; However, it is unclear whether this practice is useful in all groups of patients or not [22, 23]. Some authors showed that high-risk patients, according to pre-extubation criteria, should receive prophylactic respiratory support after extubation to prevent reintubation [22, 23]. However, we assume that early parameters after extubation could help in selecting patients who would benefit from prophylactic respiratory support. Early prediction of failure could alert the intensivist to carry out more investigations (e.g., imaging, echocardiography); manage the cause of failure (e.g., diuresis, steroids), and call for senior help. One of the important lessons from the 2019 Corona virus crisis is urging the need for vacant intensive care unit beds. Clearing the bed after extubation could help in admitting new patients and saving more lives; finding accurate and simple tools for this purpose is worth investigation. Our results introduce a novel and important use for the PPI as a non-invasive and easy tool for following-up patients after extubation.

This study has some limitations. It is a single centre study. We included a mix of surgical intensive care patients (surgical postoperative, trauma, neurological, and obstetric patients). Patients with brain pathologies commonly fail due to inadequate cough; therefore, their evaluation requires a separate study. However, in the current study, the number of patients with primary neurologic pathology is low and none of them failed due to poor cough. Therefore, it is recommended to do more focused future studies on subgroups of surgical patients as well as in medical patients to confirm the cut-off values. Another limitation is that the study lacks a validation cohort.

In conclusion, PPI early after extubation is a useful tool for predicting reintubation. Low PPI is an independent risk factor for reintubation. A PPI > 2.5, one hour after extubation could confirm successful extubation.

## Data Availability

The datasets used and/or analysed during the current study are available from the corresponding author on reasonable request.
